# Food security intervention mechanisms in the drought-prone rural areas of Tigray

**DOI:** 10.3389/fnut.2024.1413017

**Published:** 2024-08-13

**Authors:** Tewelde Gebre, Zenebe Abraha, Amanuel Zenebe, Woldegebrial Zeweld

**Affiliations:** Institute of Environment, Gender and Development Studies, Mekelle University, Mekelle, Ethiopia

**Keywords:** food policy, Safety Net, drought, rural, interventions, food aid, humanitarian assistance

## Abstract

**Introduction:**

Tigray is one of the food-insecure regions with many people living under the condition of chronic hunger. Proper intervention mechanisms are vital for addressing food insecurity. Yet, food security intervention mechanisms of various levels are not researched well. Besides, previous studies have rarely addressed the objectives of food security intervention mechanisms in relation to the four pillars of food security: availability, access, utilization, and stability. Thus, this study aims to investigate the food security intervention mechanisms in the drought-prone rural areas of Tigray in relation with the major components of food security.

**Methodology:**

This study has employed a cross-sectional study design based on a mixed research approach with primary and secondary data. For this, 363 households from three selected drought-prone rural districts, i.e., Atsbi-wenberta, Irob, and Hintalo- wejerat were studied. Primary data were collected using questionnaires and key-informant interviews. And, secondary data were collected from relevant archives and policy documents. The obtained data were analyzed descriptively and content-wise.

**Results:**

Findings show that there were several international interventions intended to halt food insecurity sustainably through financial aid, but many of the interventions were found to be responding to humanitarian crises mainly the food shortages. Ethiopia's Food and Nutrition Policy, Food Security Program, Food Security Strategy, and Food Security Pack program were the food security intervention mechanisms at the national level. These interventions were found to be inconsistent with each other in their intended goals. Regionally, no food security strategy or program was found intervening to the prevailing food insecurity in Tigray. More notably, the region has no food security bureau or office that deals with food security issues of the region. At a community level, food aid, and PSNP transfers have been the usual food security intervention mechanisms. 35.6% (77,010) of the population in the study rural districts were found to be rural PSNP beneficiaries. The food aid and PSNP transfers were outrageously insufficient for the recipients to cope with food insecurity.

**Conclusion:**

Intervention mechanisms should focus on enhancing vulnerable households' coping and adaptive capacities to deal with food security problems. In this regard, all the food security intervention mechanisms of various levels should be integrated into the common goal of achieving food security.

## Introduction

Food insecurity is a condition when all people at all times do not have social, physical, and economic access to sufficient and nutritious foods that meet their requirements (1). Several interventions have been taken to halt food insecurity from the global to household levels. As a result, considerable stride was made globally in reducing hunger between 1990–1992 and 2014–2016, when the proportion of food-insecure people reduced from 23.3 to 12.9% (2). However, the number of severely food-insecure world population increased from 7.5% in 2017 to 9.2% in 2022 (3).

The international community has shown its commitment to combat food insecurity by adopting the 2030 agenda for the Sustainable Development Goals (SDGs) in 2015, including targets to end hunger (SDG 2) and ensure access to food by all people (target 2.1); end all forms of malnutrition (target 2.2); and double the agricultural productivity and incomes of small-scale rural food producers (target 2.3) (4). Nevertheless, the number of global people facing hunger has been increasing since 2017 (3), implying the improbability of achieving the SDG target to eliminate hunger.

Further, the number of food-insecure people is still high in Eastern Africa, where nearly 30% (327.1 million people) of its population are food insecure (3). A recent assessment of food security projected that 15.8 million people in Ethiopia will face hunger and need food assistance in 2024 (5). In Tigray, food insecurity is at a critical level as millions face extreme challenges to access food in many parts of the region (6). According to Oxfam (7) report, about one million (more than 20%) people in Tigray are facing acute hunger, and 3.5 million people are in urgent need of food aid. Unless efforts are harshly stepped up, more people in the vulnerable rural areas of the region could be starved.

Given the growing number of food-insecure population and the increasing need to improve population health, there is wide interest in addressing food insecurity. The UN agencies are now calling for new ways of thinking to integrate food security concerns into different global and national development plans (8).

Achieving food security is a long-term and multi-sector process that requires a fundamental social transformation and timely financial and technical support to vulnerable households (9). In recognition of this, food security intervention mechanism is a central element in the pursuit of food security. A review of intervention mechanisms by Bizikova et al. (10) revealed that a positive impact of food security intervention mechanisms was reported in 73 publications (67% of the reviewed publications). Similarly, another review indicated that intervention mechanisms at a community level that include agricultural strategies like raising agricultural awareness, improving soil and seeds, promoting gardening, and agroecological practices were reported to have improved food security (11).

In Ethiopia, several food security intervention mechanisms are in place; however, only the PSNP and agricultural interventions were repeatedly mentioned (12–14), and other food security intervention mechanisms are not scientifically reported. Further, Van der Veen and Gebrehiwot (15) described the effect of policy intervention on food security in Tigray, but the study did not include other interventions.

To meet the ideal concept of food security (1) at all levels, intervention mechanisms should address the four basic components of food security: (1) increasing food availability at household level; (2) ensuring access to food; (3) ensuring access to safe and nutritious food; and (4) sustaining food stability. However, scholars who studied food security intervention mechanisms like Saleth and Dinar (16), Bizikova et al. (10), and Nisbet et al. (17) did not adequately report the integration of food security components into the intervention mechanisms. Therefore, this study will fill the knowledge gap by identifying the prominent food security interventions that can impact the vulnerable rural households in relation to the basic pillars of food security.

Improving food security is frequently expressed as a goal of governments, multilateral development agencies, and non-governmental organizations (18). Thus, to better understand the scope of food security interventions by governments, multilateral development agencies, and non-governmental organizations, four categories of intervention mechanisms are identified in this study: international, national, regional, and community level food security intervention mechanisms. Thus, this study aims to describe the existing food security intervention mechanisms of these various levels in relation to the four pillars of food security.

Although food insecurity intervention mechanisms need to be developed for specific contexts, this study will provide valuable lessons for successful interventions in other countries. Further, the study will serve as a base for other researchers interested in evaluating these intervention mechanisms' impacts on food security.

## Research methods

### Research design

To describe the existing food security intervention mechanisms, a mixed research approach is applied in which quantitative and qualitative studies were integrated to describe the results of the study numerically and in a meaningful way. To efficiently collect factual data from the sampled population using a questionnaire, a cross-sectional survey research strategy was employed.

### Data type and sources

This study is based on primary and secondary data, in which household heads, PSNP officers, and relevant policy archives were the data sources.

### Sampling techniques and study population

This study has used both probability and non-probability sampling techniques; from the non-probability sampling techniques, a judgmental sampling method was used to select rural districts and the target population. Based on the purposes of the study, drought-prone rural areas with a higher proportion of food-insecure population were selected to take sample participants for the study.

**Figure 1:**
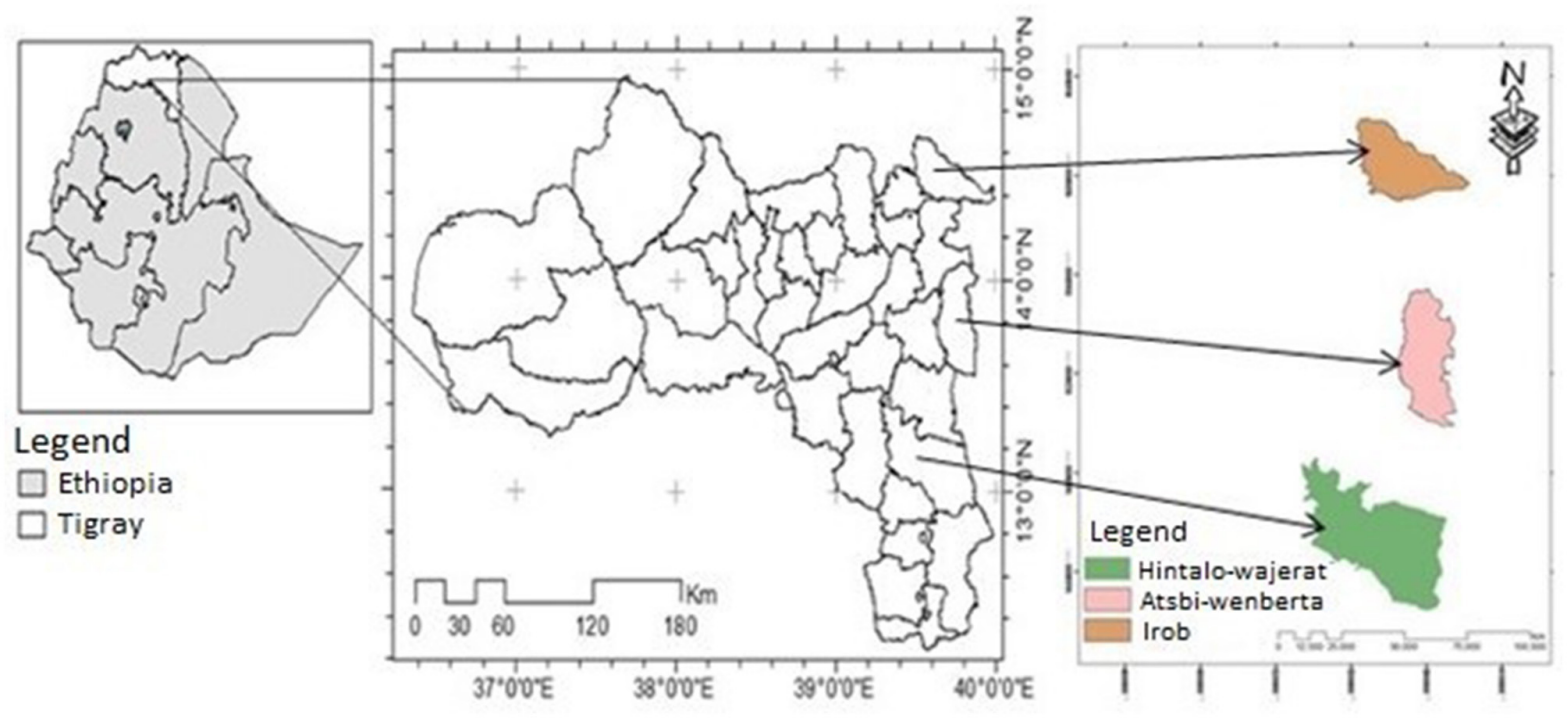
Administrative map of the study areas.

During 2021, data obtained from the Tigray region office of food security shows that *Irob, Atsbi-wenberta*, and *Hintalo-wejerat* rural districts, depicted in Figure 1, were the most drought-prone rural areas of Tigray region with a higher proportion of food-insecure population (19). And, two sub-districts (tabias) from each district with a higher number of food-insecure population (based on district's PSNP data), six sub-districts in total were selected to conduct the survey. Hence, *Alitena* and *Haraze-sebeata* from *Irob* district; *Haresaw* and *Hadinet* from *Atsbi-wenberta* district; and *Bonka* and *Seneale* from *Hintalo-wejerat* district were selected for this study.

Rural Productive Safety Net Program (PSNP) beneficiaries were the target population for the study. In these stated sub-districts, there were a total of 6,528 food-insecure households being benefited by rural PSNP. This number was considered for drawing the final sample size. To draw the sample size, the following formula was adopted from the Survey Monkey (2023), which is the easiest and faster online sample size calculator for survey researches. The formula is based on margin of error and confidence level, and is convenient for a finite population.


$$\begin{array}{l} n=\frac{\frac{z^{2*}p\left(1-p\right)}{e^{2}}}{1+\left(\frac{z^{2*}p\left(1-p\right)}{e^{2}N}\right)} \end{array}$$

Where *n* = sample size; *N* = population size; *e* = Margin of error; and *z* = confidence level.

Assuming a 95% confidence level and 5% margin of error, the final sample size was found to be 363. The final sample size was distributed to each of the sub-districts proportionally as depicted in Table 1. By the end of the survey, all the filled-in questionnaires were collected.

**Table 1:** Distribution of the sample size to sampled study areas.

**No**	**Name of the sub-district**	**Number of food-insecure households**	**Final sample size**
1	*Alitena*	1,029	57
2	*Haraze-sebeata*	1,174	65
3	*Gonka*	1,022	56
4	*Seneale*	1,235	69
5	*Haresaw*	1,080	61
6	*Hadinet*	988	55
7	Total	6,528	363

Further, a simple random sampling was employed in the selection of respondent households for administering the questionnaire. This is mainly because of the availability of organized data on the number of rural PSNP beneficiaries.

## Methods of data collection

### Questionnaire

Data regarding the food security intervention mechanisms at community level were collected through an interview-based questionnaire. Both open and close-ended questionnaires were administered to selected respondents. This is to obtain structured responses and self-expressed opinions from the household heads. The questionnaires were translated into the local language (Tigrigna and Saho) and administered through face-to-face interaction with household heads by trained enumerators (Food security experts of the respective study areas).

Prior to the field survey, the questionnaires were tested through a pilot survey in selected 20 households, which was conducted out of the selected study areas. Lastly, the tool was administered after possible improvement of interview questions was successfully conducted and checked against any errors after the pilot testing.

Since the questionnaires were filled in by trained enumerators, all the administered questionnaires were returned with minimum acceptable errors; and, the errors were corrected in field. Finally, the data were carefully coded and data entry was followed using SPSS V. 27.

### Key informant interview

Key informant interview was used to gather food security intervention mechanisms at regional and community levels. A semi-structured interview was employed to acquire data from head of the Tigray region PSNP office. This is essentially to make the interview guided by flexible schedules for asking elaboration and to make the interviewee more relaxed.

### Documentary sources

Relevant policy documents including Ethiopia's Food Security Policy, Food Security Strategy, Food Security Program, PSNP implementation manual were analyzed to describe the existing food security intervention mechanisms at various levels.

### Data analysis techniques

Qualitative data were mostly obtained from documents regarding food security intervention mechanisms. Hence, a qualitative method of data analysis, i.e., documentary and content analysis were mainly used for analyzing the data that were obtained from the relevant documents. And, a quantitative method of data analysis (descriptive analysis) was used for analyzing the data obtained from the questionnaire regarding intervention mechanisms at the community level. Further, a narrative analysis method was applied to the data obtained through the interview.

### Ethical considerations

The study has no ethical issues related to animal and human rights. The anonymity of respondents and the identity of the researcher were considered as ethical issues in this study. Thus, the researcher/data enumerators took an official letter from the university to handle any uncertainties during the field survey. The letter was used to make clear to the data providers that the data was only to be used for research purposes.

Besides, the researcher/data enumerators have asked the respondents their consent to participate before the data gathering, and the anonymity of the respondents was ensured. Moreover, politeness and courtesy in gathering the required data were the ethical guides for the researcher/data enumerators.

## Results and discussion

### Interventions at international level

Finance is a major enabler for sustainable agricultural growth and therefore food security in Ethiopia. External aid, on this regard, one of the ways that the international community can contribute to the wellbeing of humans. Ethiopia is one of major recipients of international assistances. Ethiopia was receiving external aids since the late 1940s (20). Based on foreign aid and official development assistance received from 1960 to 2021, Ethiopia was ranked fifth out of 134 countries and second in Africa (21). This shows Ethiopia has been attracting international assistances to support its food security achievement activities and this can be taken as a good opportunity for the country.

Food is one of the main sectors attracting international interventions in Ethiopia. During 1960–2003, food was the major sector that received the largest aid money in Ethiopia (22). In 2012, Ethiopia was the top food aid recipient country in the world accounting for 16% of global food aid receipts (23). According to OCHA (24), USD 572.6 and 175 million was internationally donated to Ethiopia in 2023 to the food and nutrition sector, respectively to respond to the food crises.

In addition to the national efforts, the international donor community, NGOs and the United Nations have been contributing to the food security status in the affected populations in Ethiopia. Based on the analysis of food-related international contributions to Ethiopia, the European union was found to be the major contributor to food-related activities in Ethiopia. Similarly, Lemi (22) indicated that the European Union was major multilateral donor to Ethiopia during 1960–2003 followed by the World Bank and World Food Program. The European Union was supporting food-insecure households by providing food and non-food items distributed through ECHO (European Community Humanitarian Office) channeled by the Relief Society of Tigray (REST) and the International Committee for the Development of People (CISP) in Tigray and Afar Regions.

Besides, the governments of Britain, Italy, Germany, Ireland, Norway, USA, Japan, France, Netherlands, and Switzerland have been the long-time consistent contributors to food-related activities in Ethiopia. Further, Belgium, Finland, Canada, Sweden, Spain, and Austria have been also contributing for food-related activities in Ethiopia in an irregular manner. According to Lemi (22), USA, Italy, Germany, Sweden, and the Netherlands were the top donors in that order during 1960–2003. In 2023, USA, Germany and Canada were the top contributors to Ethiopia humanitarian response plan (24). This indicates Ethiopia can have secure financial supports in its effort to achieve food security.

On the other hand, the WFP has been an important UN-agency for allocating food items in Ethiopia. During March 2021–May 2022, WFP has reached 4.4 million food-insecure people in Northern Ethiopia (25). WFP has been using TSFP (targeted supplementary feeding program) and BSFP (blanket supplementary feeding program) nutrition activities to treat and prevent moderate acute malnutrition for 47,000 under 5 years of age children and pregnant and lactating women in Tigray region in February 2022 (25). Although this activity cannot contribute to the sustainable food security of the affected population, it is important that the WFP was focusing on nutrition beyond the food availability.

A review of international interventions to food insecurity reveals that many international donors preferred to respond to food insecurity by providing direct donations to implementing agencies. Nonetheless, there have been some substantial food aids in the late 1990s and early 2020s. The review shows that displaced people were major recipients of the food and non-food aids.

Apart from food aid supplies, a promising international intervention was identified in supporting the Ethiopia's rural Productive Safety Net Program (PSNP). Unlike the food aid supplies, the rural PSNP aims at addressing the chronic food insecurity in selected vulnerable communities. According to World Bank (26) phase four implementation report, the program has reached nearly eight million people in 382 food-insecure rural districts of Ethiopia. However, there is no clear data about what food security components the program is addressing and how.

Several development partners were active in financing the rural PSNP. A total of USD 3,021,019,353 was incurred for the implementation fourth phase of the program; of which 84.3% of the cost was financed by international donors. According to Table 2, the World Bank was the major donor of the program followed by DFID and USAID.

**Table 2:** Multilateral donations to PSNP fourth phase.

**No**	**Name of donors**	**Actual amount disbursed (USD)**
1	World Bank	1,623,979,010
2	USAID	360,000,000
3	DFID (British Department for International Development)	502,953,948
4	Ireland Government (Irish Aid)	58,601,763
5	UN children's fund	16,464,689
6	World Food Program	29,990,990
7	Australian Development Agency	2,216,601
	Total	2,547,751,322

Source: World Bank (26).

After the completion of the fourth phase of RPSNP, the fifth phase of the program was launched with a total of USD 2.2 billion investment to reach up to nine million food-insecure people (27). This shows the international communities are ready to contribute for the achievement of food security in Ethiopia. Yet, there are lack of clarities on how the rural PSNP can contribute to the four components of food security through the five sub-programs: public works, temporary direct support, permanent direct support, livelihoods services, and shock responsiveness.

The Ethiopia's Agricultural Growth Program (AGP) has been the other major program where international financial intervention is visible to see. The program aims at enhancing agricultural productivity and access to market with active participation of the women and youth partly meeting the food availability and access to food pillars of the food security. According to GAFSP (28), nearly 700,000 farmers were benefited from AGP–I.

The first phase of the program's total cost was USD 417,800,000 (29); of which 94.8% was financed by international multilateral and bilateral donors. Based on Table 3, the World Bank was the major donor to the implementation of AGP-I.

**Table 3:** International donations to AGP-I.

**No**	**Name of donors**	**Actual amount disbursed (USD)**
1	World Bank	256,200,000
2	USAID	81,400,000
3	UN Development Program	2,400,000
4	Other bilateral agencies	56,200,000
	Total	369,200,000

Source: IED (29).

FAO, unlike the other supporters, have been providing technical support for effective implementation of the Ethiopia's rural PSNP. It has also been supporting the adaptation strategies to agricultural drought in Ethiopia through strengthening institutional capacity for resilience; supporting early warning and information management systems; building community level resilience; supporting communities through diversified livelihood options; and supporting irrigation development activities (30). This initiative promotes achievement of food security by enhancing agricultural productivity in general and food availability particularly.

Although there were several international interventions intended to halt food insecurity sustainably through financial aids, much of the interventions were found to be responding to humanitarian crises mainly the food shortages. This implies that due focus was given to the transitory food insecurities. According to OCHA (24), out of the planned USD 3.995 Billion, USD 1.339 Billion were received and financed to respond to the food crises in Ethiopia in 2023. If these were to be financed to initiatives that work on achieving sustainable food security, the impact of these finances would have been much greater.

WHO (31) reported that, “*Investing one dollar per person per year could save seven million lives in low- and lower-middle-income countries*”. Similarly, the National Institute of Building Sciences (32) reported that every USD one invested in disaster mitigation saves extra responding costs of USD six. This shows a significant savings can be gained by investing in preparation and mitigation works. Further, proactive measures can contribute to achieving food security by increasing agricultural productions and strengthening households' capacity to withstand shocks.

### Interventions at national level

Public sector intervention mechanisms have a prominent role in addressing food insecurity at household level. National policy is one of the fundamental ways to guide intervention mechanisms to food insecurity and its associated causes.

The FDRE (33) “Food and Nutrition Policy” was the first national food and nutrition policy in Ethiopia. The policy emphasizes on attaining optimal nutritional status of all age groups at all levels. The policy has generally seven directions to achieve its intended objectives:

- Ensuring food availability, accessibility and utilization of diversified, safe and nutritious foods in a sustainable way;
- Ensuring the safety and quality of foods from production to consumption;
- Improving postharvest management of farm products;
- Ensuring optimum nutrition at all age groups;
- Providing timely and appropriate emergency response for food crises;
- Strengthening food and nutrition communication; and
- Establishing and strengthening food and nutrition governance.

The food and nutrition policy of Ethiopia is generally found to be well-elaborated and logically linked to the four major components of food security. The policy is formulated with a broader scope and provides a base for multi-sectoral collaboration, interventions and planning food security strategies. Nevertheless, the policy is found to be more sensitive to nutrition than the other pillars of food security. In addition, the rural people in general and the vulnerable food-insecure rural people in particular were not given appropriate emphasis in the policy.

Unlike the Food and Nutrition Policy of Ethiopia, the Food Security Program of Ethiopia exclusively emphasizes on vulnerable rural people. According to the Ministry of Agriculture and Rural Development (34), the long-term goal of the Food Security Program of Ethiopia has been: achieving food security for the chronic and transitory food-insecure rural households in Ethiopia. Nevertheless, the program has no clear ways of addressing the pillars of food security.

The Food Security Program of Ethiopia has been under implementation since 2003 in 319 chronically food-insecure rural districts of Ethiopia with four distinct components: Productive Safety Net Program (PSNP), Household Asset Building Program (HABP), Complementary Community Investment program (CCI), and resettlement program.

PSNP has been intended to provide transfers through labor intensive public works for those who are able bodied and direct support for those who are unable to work. According to Ministry of Agriculture (35), the fifth PSNP has five sub-programs: public works, temporary direct support, permanent direct support, livelihoods services, and shock responsiveness. During phase one and two, there were nearly 4.8 million PSNP beneficiaries (36). And, according to World Bank (26), there were 7,997,218 PSNP country-wide beneficiaries in 2022; of which 2,770,188 were under direct support of the program.

PSNP was first launched in 2005 and it is now in its fifth phase (27). The fourth phase of the PSNP was extended three times in 2020 and 2021, for a total of 18 months: from December 2020 to June 2021; June 2021 to December 2021; and December 2021 to June 2022 (26). The fourth phase of the program was ended in June 2022; and its project performance was rated as “moderately satisfactory” by the World Bank (26). In 2016, urban PSNP was launched and started to be implemented in 11 major urban areas of Ethiopia (37).

Phase five of PSNP focuses on eliminating extreme poverty in drought-prone areas. Its target shifted from chronically food insecure households to extremely poor households, thereby targeting and addressing the needs of the extremely poor and the most vulnerable. While focusing on drought-prone areas is a good direction to reach the vulnerable households, focusing on extreme poverty exclusively may not address the multi-faceted root causes of food insecurity. This is mainly because food insecurity in rural Ethiopia is mostly caused by low productivity in the rainfed agriculture (38).

HABP aims at building productive assets and diversifying income sources of the food-insecure households. The interventions include disseminating improved agricultural inputs, moisture conservation and utilization techniques, and farmers trainings for generating additional incomes. CCI, on the other hand, aims at improving food-insecure households' access to basic social services and infrastructures.

The resettlement program, unlike the other components, aims at ensuring food security of vulnerable households by providing an access to farmland in other areas. The food-insecure households must move to other areas for accessing the farmland. The settlers are provided with basic agricultural inputs and food assistance until their first production.

The food security program of Ethiopia is generally reasonable that it gives due focus to the chronically food-insecure rural households. More importantly, the program has set to at least achieve food availability at household level, if achieving food security failed for any reason.

The limited emphasis given to nutrition aspect is one major drawback of the program. The program hardly show how nutritional security can be achieved in rural Ethiopia. Besides, food security in the program is viewed as a “relative” state of resilience than the globally accepted working concept. Further, the program lacks clarity in both the concept and standards of “graduation” of food-insecure households. The program set stable livelihood and increment in asset buildings as a requirement for graduation; but no method was designed to measure the requirements.

Ethiopia's Food Security Strategy is the other food-insecurity intervention mechanism at national level. The strategy was first formulated in 1996 and updated in 2002. The FDRE (39) Food Security Strategy of Ethiopia relies on three basic pillars to achieve food security: increasing food availability through enhanced domestic production; ensuring access to food in food-insecure areas; and strengthening emergency response capacities. The strategy targets mainly the chronically food-insecure households living in drought-prone and pastoral areas.

Unlike the Food and Nutrition Policy and Food Security Program, the Food Security Strategy intends to address the causes of food insecurity in Ethiopia. Accordingly, environmental rehabilitation, water harvesting schemes, livestock and agro-forestry development, and cultivation of high value crops were given a clearer focus on the strategy. In addition, the strategy explains that there are linkages between chronic and transitory food insecurity in that unless households were chronically food-insecure, unpredictable shocks cannot rapidly lead to transitory food insecurity. This is a good initial to address the stability component of food security.

What is so special about the Food Security Strategy of Ethiopia is that, food insecurity is categorized in to three categories: rural food insecurity, urban food insecurity, and others (displaced people and groups affected by instability). More importantly, it is highlighted that the causes to chronic and transitory food insecurity in these three categories are distinct. The various regions were also described based on poverty measures to indicate their susceptibility to food insecurity. This has a vital significance in guiding food-security interventions at local levels.

Generally, good qualities the Food Security Strategy of Ethiopia is that it explains the importance of understanding the nature of food security and coping mechanisms adopted by vulnerable households. Moreover, it states that addressing the root causes of food insecurity is an instant requirement to achieve food security. Key to this, boosting agricultural production, building the resource base of chronically food-insecure households, diversifying income sources in both rural and urban areas, and providing transfers to targeted households are proposed as ways of achieving intended outcomes of the strategy.

Nonetheless, like the Food Security Program of Ethiopia, less emphasis was given to the nutrition aspect in the Food Security Strategy of Ethiopia when compared to the other components of food security. In addition, much less emphasis is given to the small farm size households living in drought-prone rural areas. The strategy directs a resettlement program for small farm size food-insecure households living in drought-prone rural areas. Given the positive effect of resettlement program on food security, the strategy should have indicated alternative interventions for those households who are unable to resettle in other areas.

Concerning the food-insecure rural households living in drought-prone districts, the Food Security Pack (FSP) program was introduced in 2000 by Ethiopian government with aim of empowering vulnerable but viable farmers affected by recurrent droughts (40). The program has three major components namely: (1) Rainfed Cropping in which farmers are supported with fertilizer and seeds for two consecutive years; (2) Wetland Cropping in which farmers are supported with fertilizer and seeds for one cultivation season only; and (3) Alternative Livelihood Initiative in which farmers are supported with non-crop agricultural inputs like goats, sheep, and chickens.

According to MCDSS (40), the FSP program has been under implementation country-wide in selected 116 districts. The Alternative Livelihood Initiative of the program is a good opportunity to enhance food security of physically disabled and small farm size households living in drought-prone areas. However, it is flawed to see that the two components of the FSP program targets only able-bodied farmers with minimum farm size of 0.5 hectares. Thus, attainment of the social access to food is in question.

### Interventions at regional level

The FDRE (39) Food Security Strategy dictates that food security interventions at regional level should be designed on the basis of the Food Security Strategy of Ethiopia. Based on Van der Veen and Gebrehiwot (15), the food security interventions in Tigray were giving utmost attention to agricultural extension services that includes the use of fertilizers and improved seeds, facilitating farmers' access to rural credit, introducing better and improved agricultural practices, and introducing a variety of water harvesting schemes. These interventions significantly contributed to a higher likelihood of household food security status in Tigray over the period of 2000–2008 (15).

Soil and water conservation activities in Tigray have been one of the widely practiced interventions in the region. Until the 1960, there was no soil and water conservation programs in Tigray, and the first conservation practices at hillside scale took place in the early 1970s (41). Since 1985, soil and water conservation activities has been implemented in Tigray in wider scale (42). In September 2017, Tigray was awarded a gold by the World Future Policy for restoring land on a massive scale. A focused soil and water conservation interventions would contribute to the region's rural food availability and access by enhancing agricultural productivity.

Apart from PSNP and some ongoing projects including the WFP and FAO led food security related projects, no regional strategy or program was found that deals with the rural or general food security issues of the region until February 21, 2024. All the regional interventions were guided by the national food security policy, program and strategy. Given that more than half part of Tigray region is dry-land (43) and thereof higher vulnerability of the rural people to food insecurity, region specific food security programs and strategies would have greater importance in guiding food security intervention mechanisms.

More importantly, no regional food security bureau or office that specifically deal with food security issues was found during the survey. The PSNP office was acting as food security office of the Tigray region. At federal level of Ethiopia, there were Disaster Risk Management and Food Security Sector under the National Disaster Risk Management Commission (NDRMC) until 2015; the Food Security Sector was later shifted to Ministry of Agriculture and Natural Resources to deal with the PSNP (26).

In Tigray region, there were Disaster Risk Management (DRM) Directorate and Food security sector under Bureau of Agriculture and Natural Resources of Tigray region. The Disaster Risk Management Directorate was responsible for coordinating the disaster management and implementation of the Humanitarian Food Assistances (HFA); and, the food sector was responsible only for the PSNP.

Hence, the PSNP office was found as the only regional office related to chronic food insecurity issues in Tigray region. According to data obtained from the PSNP office during January 2024, there were a total of 1,010,752 rural PSNP beneficiary households in Tigray; of which 247,945 households were under the direct support program (Woldelibanos D., personal communication, January 16, 2024). This accounts for 12.6% of the total national PSNP beneficiary households in Ethiopia.

More importantly, according to the head of Tigray region's PSNP office, out of the 34 rural districts, there were a total of 31 rural districts under the benefits of rural PSNP in 2019; and, following the administrative boundary restructuring that took place in 2020, the PSNP rural districts increased to 56 (out of the 57 total rural districts in the region). Accordingly, PSNP beneficiaries in rural districts of Tigray increased from 91% in 2019 to 98.2% in 2022.

HFA is the other widely practiced food security intervention in Tigray. In the region, HFA was intended for non-PSNP households who are affected by the transitory drought (44). HFA and PSNP are coordinated in a way that HFA targets those who are temporarily (transitory) food-insecure and PSNP targets chronically food-insecure; hence, there would be no overlap.

By the end of 2023, there were a total of 340 local and international humanitarian agencies registered in Tigray region, according to Bureau of Social Affairs of Tigray. However, no data was available regarding which of these humanitarian agencies were dealing with food security issues of the region. During 2021, there were only ten international NGOs, three national NGOs, five UN agencies, the International Committee of the Red Cross (ICRC), and two donor entities operating inside Tigray (45).

### Interventions at community level

Community-level interventions play a vital role in addressing societal problems. A study by Durao et al. (46) showed that a community-level interventions had improved food security status in low and middle-income countries of Africa and Latin America. Similarly, Doustmohammadian et al. (11) have reviewed the impact of community-level interventions on food security and its dimensions. The authors reported that all community-level interventions have significantly contributed to the intended food security targets.

Food aid has been one of the food security intervention mechanisms at community level. According to Stoddard et al. (45), food was the most needed and received type of aid in Tigray followed by medicines. OCHA (24) reported that nearly 1.2 million households have received food aid in Tigray in 2023.

The food-insecure rural households living in the selected drought-prone rural areas of Tigray were asked if they received food aid during May 2022 to April 2023. Accordingly, 344 (94.8%) of the respondents claimed that they have received food aid during that time; however, almost all (96.2%) the food aid recipients revealed that the food aid was not sufficient to cope with their food insecurity, as illustrated in Table 4. Stoddard et al. (45) reported that 94% of surveyed Tigray people stated that they have been in need of aid since November 2020, but only 43% reported receiving food aid during 17 February to 8 March of 2021.

**Table 4:** Questions related to food aid at the study areas.

**No**	**Questions**	**Yes**		**No**	
		**Frequency**	**Percent**	**Frequency**	**Percent**
1	Have you received food aid during the last year?	344	94.8	19	5.2
2	Was the food aid sufficient?	13	3.8	331	96.2

**Figure 2:**
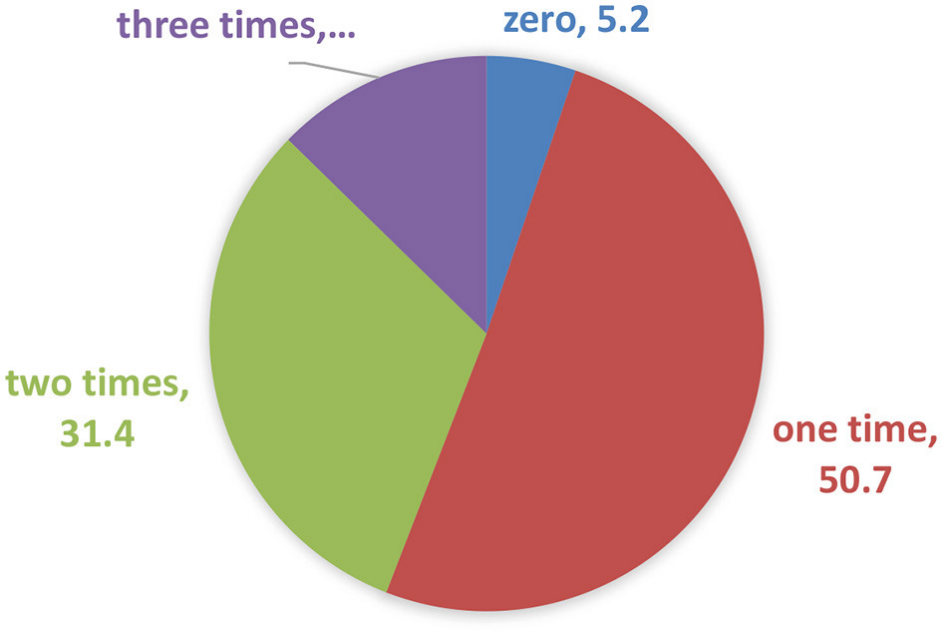
Frequency of food aid received by the food-insecure households (1st May 2022–30 April 2023).

The OCHA (24) report additionally indicated that the food aid in Tigray was irregular and 61 districts, including the drought affected areas were reached with in-kind and cash-based assistance. In line with this, Figure 2 shows that more than half of the food aid recipients reported that they had received food aid only once; while 12.7% of the households had received the aid three times during May 2022–April 2023.

Rural PSNP is the other widely practiced food security intervention mechanism at community level. According to the program implementation manual designed by Ministry of Agriculture (35), the number of PSNP beneficiaries is determined by the established quotas per districts and sub-districts, and not all households that meet the eligibility criteria are selected.

In the selection process, households are compared to each other and ranked based on the socio-economic status including land and livestock holding; and people with disabilities, female-headed households, households with members suffering from chronic illness, elderly headed households caring for orphans are prioritized. Subsequently, households are assigned to either the public works or to the direct support components based on their household characteristics and physical capability to work. This enhances the economic and social access to food.

Rural PSNP beneficiaries are reassessed annually to determine their eligibility to stay supported. If socio-economic condition of beneficiaries is improved or if they have been in the program for at least 3 years, certification is done. Certified households will be replaced by other selected vulnerable households. In line with this, Demsash et al. (47) reported that poor households in Ethiopia were 1.9 times more likely to receive PSNP benefits than rich households; and, rural households in Ethiopia were 2.2 times more likely to receive PSNP benefits than urban households.

Rural PSNP is the dominant food security intervention mechanism in Tigray and the study areas in particular. Table 5 shows there were a total of 77,010 (35.6% of the population) rural PSNP beneficiaries in the three drought-prone rural districts in December 2023. Of which, 20,019 (25.9% of the beneficiaries) were direct beneficiaries and the rest were public (food for work) participants, as shown in Table 5.

**Table 5:** Rural PSNP beneficiaries in the study areas (December 2023).

**PSNP beneficiaries**	**Atsbi**	**Irob**	**Wejerat**	**Total**
Total population (as of July, 2023)	94,210	33,280	88,800	216,290
Direct beneficiaries	6,851	7,664	5,504	20,019
Public work beneficiaries	23,526	20,003	13,462	56,991
Total rural PSNP beneficiaries	30,377	27,667	18,966	77,010
Percentage of beneficiary/ population	32.2	83.1	21.3	35.6

According to the food security team leaders of the respective rural districts, the transfer was ETB 225 per month up to June 2023; it was later raised to ETB 420 (USD 7.434, as of January 01, 2024)^1^ starting from July 2023. The transfer was not only much less to support households access to food, but also it does not ensure that the purchasing power of the transfers keeps pace with food price changes. This is supported by studies made by Desalegn and Ali (48) and Hirvonen and Hoddinott (49).

Furthermore, there were quite contradicting findings concerning the impact of PSNP on food security status of beneficiary. A country level study on the impact of PSNP found that PSNP reduces the initial impact of drought by 57%; and it reduces the persistence of post-drought impacts from 4 to 2 years (50). In addition, a review on the impact of PSNP by Desalegn and Ali (48) showed that PSNP had generally a positive impact on food security of households. Similarly, Tadesse and Gebremedhin (51) indicated that PSNP has enhanced consumption expenditure, daily calorie intake and annual income of participating households in Gedeo zone of Ethiopia. Further, similar study in Somali region of Ethiopia reported that PSNP had a positive and significant impact household's food security status; and participants of PSNP were reported to have more daily calorie intakes than the non-participants (52).

In contrast, Demsash et al. (47), reported that PSNP was not effective in ensuring food security or children dietary diversity in Ethiopia. Similarly, Bahru et al. (8) reported that PSNP has not improved household food insecurity, child dietary diversity, and child malnutrition in Ethiopia. Mustafa et al. (53) also reported that PSNP had only increased current consumption pattern, and graduated households had to return to their previous food insecurity situations. Furthermore, Bahru and Zeller (36) reported that no evidence was found to ensure that PSNP has improved households' agricultural technology adoption, time spent in agriculture, household's access to agricultural services, and women's asset ownership.

## Conclusion

Food has been one of the main sectors attracting international interventions in Ethiopia since 1960s; Ethiopia has been the top food aid recipient country in the world. A number of the international donor community, NGOs and the United Nations have been contributing to the food security status in the affected populations in Ethiopia mainly in the form of food aid.

Although there were several international interventions intended to halt food insecurity sustainably through financial aid, many of the interventions were found to be responding to humanitarian crises mainly the food shortages. Rural PSNP, which has been under implementation since 2005, was predominantly funded by the international multilateral and bilateral donors. Nearly half a billion is funded annually to the PSNP implementation by international donors. Yet, more proactive measures that increase agricultural production and strength households' capacity to withstand shocks are much needed to achieve food security.

At the national level, Ethiopia's Food and Nutrition Policy, Food Security Program, Food Security Strategy, and Food Security Pack program were the food security intervention mechanisms. The Food and Nutrition Policy is found to be nutrition sensitive with lesser emphasis given to the vulnerable rural people. In contrast, the Food Security Program which consists of the PSNP, Household Asset Building Program, Complementary Community Investment Program, and resettlement program, mainly focuses on achieving food security for the chronic and transitory food-insecure rural households in Ethiopia. Similarly, the Food Security Strategy targets mainly the chronically food-insecure households living in drought-prone and pastoral areas. Yet, nutrition was the missing aspect in both the Food Security Program and Food Security Strategy of Ethiopia. Thus, more tasks are required to harmonize the end goals of the national food security intervention mechanisms.

In Tigray region, apart from PSNP and some ongoing non-governmental projects, no regional food security strategy or program was found. More notably, the region has no food security bureau or office that deals with food security issues of the region; it is the PSNP office in charge of regional food security issues. This shows for limited political commitment of the regional government to sustainably address food insecurity when compared to earlier times. Thus, formulating a responsive and contextual regional food security policy or integrating food security into the other sectoral plans should be given due attention to address food insecurity in Tigray.

At a community level, food aid and PSNP transfers have been the usual food security intervention mechanisms. The food aid and PSNP transfers were insufficient to cope with food insecurity. Further, relying on food aid to curb food insecurity in rural areas can never address the problem sustainably. Therefore, it would be better to tackle not the symptoms, but the root causes of food insecurity by promoting enhanced agricultural and non-agricultural practices through engaging the rural households.

In general, few of the intervention mechanisms were giving considerable emphasis to the drought-prone rural areas. And, the four pillars of food security were not fully integrated in many of the international, national, regional, and community level interventions. For this purpose, future researchers have to come up with clear insights of the impacts of these interventions on food security and a critical analysis of the pros and cons of the current food security intervention mechanisms to better guide actions to address food insecurity.

## Data availability statement

The original contributions presented in the study are included in the article/supplementary material, further inquiries can be directed to the corresponding author.

## Author contributions

TG: Writing – original draft. ZA: Writing – review & editing. AZ: Writing – review & editing. WZ: Writing – review & editing.

## Footnotes

^1^One USD = ETB 56.477.
